# Mechanistic Insights into the Link between Obesity and Prostate Cancer

**DOI:** 10.3390/ijms22083935

**Published:** 2021-04-11

**Authors:** Bamidele A. Adesunloye

**Affiliations:** Cancer Treatment Centers of America, 600 Celebrate Life Parkway, Newnan, GA 30265, USA; bamidele.adesunloye@ctca-hope.com

**Keywords:** obesity, prostate cancer, adipose tissue, chronic inflammation, cytokines, adipokines, insulin-like growth factor

## Abstract

Obesity is a pandemic of increasing worldwide prevalence. There is evidence of an association between obesity and the risk of prostate cancer from observational studies, and different biologic mechanisms have been proposed. The chronic low-level inflammation within the adipose tissue in obesity results in oxidative stress, activation of inflammatory cytokines, deregulation of adipokines signaling, and increased circulating levels of insulin and insulin-like growth factors (IGF). These mechanisms may be involved in epithelial to mesenchymal transformation into a malignant phenotype that promotes invasiveness, aggressiveness, and metastatic potential of prostate cancer. A thorough understanding of these mechanisms may be valuable in the development of effective prostate cancer prevention strategies and treatments. This review provides an overview of these mechanisms.

## 1. Introduction

Obesity is a chronic and complex medical condition that is associated with excessive body fat. The risk factors are environmental, psychosocial, neuroendocrine, and genetic. Ultimately, it is the result of a long-term imbalance between energy intake and expenditure. The World Health Organization estimated that 39% of adults were overweight and 13% were obese in 2016 worldwide [1]. Obesity is a pandemic which has shown an increasing prevalence in many countries over the past four decades [2]. An analysis of the National Health and Nutrition Examination Survey showed an increase in the age-adjusted prevalence of obesity among adults in the United States from 33.3% in 2007/2008 to 39.6% in 2015/2016 [3]. The projection is that almost half of United States adults will be obese by 2030 if the trend should continue [4]. Obesity is a recognized risk factor for multiple cardiovascular, metabolic, respiratory, and musculoskeletal disorders. Multiple epidemiologic studies have also established a link between obesity and the risk of cancers, as well as cancer-related mortality [5,6]. Estimates from United States Cancer Statistics data suggest that overweight and obesity are associated with thirteen different types of cancer, and these cancers make up 40% of all cancers diagnosed in the United States in 2014 [7]. Additionally, being overweight or obese may increase the risk of aggressive forms of prostate cancer, male breast cancer, head and neck cancer, and non-Hodgkin lymphoma [8]. Multiple hypotheses have been advanced for the role of excessive body fat in carcinogenesis, clonal evolution, and drug resistance. The mechanisms are based broadly on changes in the adipose tissue microenvironment that favor induction of fibrosis and angiogenesis, stem cell abundance, expansion of proinflammatory immune cells, as well as systemic production of metabolic and inflammatory mediators. Some of the postulated hypotheses include chronic low-level inflammation among obese people, increased circulating levels of insulin and insulin-like growth factor-1 (IGF1), excessive production of adipokines and estrogen by adipocytes, and the effect of adipocytes on cell growth regulators such as mTOR and AMPK [9,10].

Prostate cancer is one of the leading cancer diagnoses worldwide and in the United States of America. In 2015, about 1.6 million cases of prostate cancer were diagnosed globally while it is estimated that 248,530 cases will be diagnosed in the United States alone in 2021 [11,12]. There was a gradual decline in the age-adjusted incidence of prostate cancer in the United States between 1990 and 2015. On the contrary, the incidence rate rose globally within the same period [11]. The proof of association between excessive body fat and prostate cancer is mainly from observational cohort and case–control studies with conflicting results. However, multiple meta-analyses have shown that there is a link between excessive body fat and prostate cancer, especially the aggressive variant [13,14]. At the cellular level, the commonly proposed mechanisms of prostate cancer tumorigenesis involve the insulin and IGF axis, deregulated adipokine signaling, and the expansion of adipose/stromal stem cells population [15,16]. The knowledge of these proposed mechanisms is still evolving. This review provides an overview of the adipose tissue as an active secretory organ, and the role of its products (secretome) and their mechanisms in prostate cancer tumorigenesis.

## 2. The Adipose Tissue

**Figure 1:**
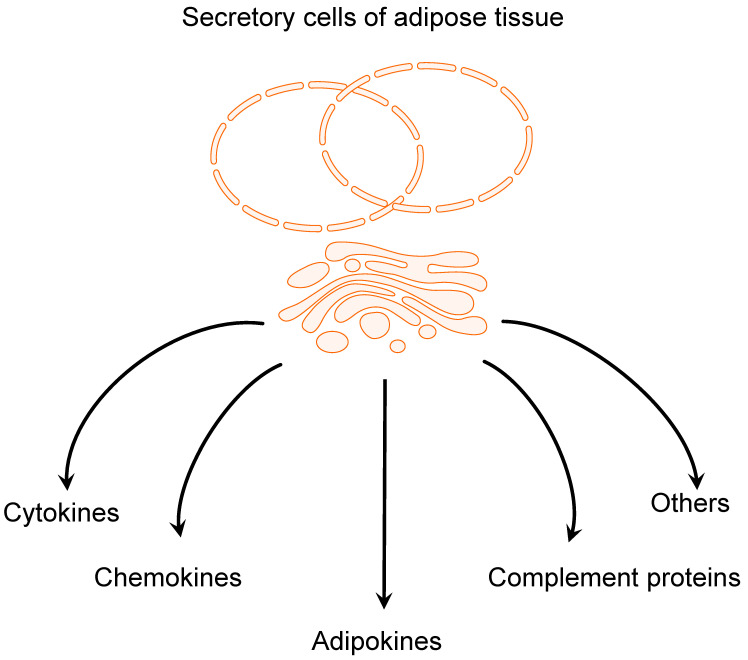
Adipose tissue secretome.

The adipose tissue is a loose connective tissue that is in distinct areas of the body called adipose depots. It has a unique ability to change its size and dimension in response to nutritional demands. The two main types are the white adipose tissue and the brown adipose tissue, which are different morphologically and in their biologic functions. In addition to adipocytes, the adipose tissue microenvironment also contains vascular endothelial cells, pericytes, fibroblasts, stem and progenitor cells, and several immune cells such as macrophages [17]. The white adipocytes are larger in size, but they contain fewer mitochondria than brown adipocytes. The white adipocyte contains a single (unilocular) lipid droplet that consists of triglycerides and accounts for more than 90% of the cell volume [18]. In contrast, the brown adipocyte contains triglyceride in multitudes of small (multilocular) lipid vacuoles and abundant mitochondria. The mitochondria express uncoupling protein 1 (UCP1), which is involved in non-shivering thermogenesis [18]. Brown adipose tissue is highly vascularized and innervated. Its brown color is attributable to the high mitochondrial density and vascularity. While the white adipose tissue serves as a store for excess energy, the brown adipose tissue is involved in heat production. Although most of the fat depots are of the white adipose tissue subtype in adults, brown adipose tissue predominates in neonates due to its function in thermogenesis, presumably to provide the much-needed insulation required at birth [19]. The white adipose depots are mainly subcutaneous and visceral. Subcutaneous adipose depots are in the mammary, abdominal, gluteal, and femoral areas. Visceral adipose tissue surrounds vital internal organs, including the prostate. There is increasing evidence that far from being biochemically inert, the white adipose tissue is an endocrine and secretory organ that plays an important role in hematopoiesis, lymphopoiesis, inflammation, reproduction, and the immune system (Figure 1). These functions are mediated through the production of hormones or cytokines [20]. Obesity is an expansion of the adipose tissue due to hypertrophy of pre-existing adipocytes or recruitment (hyperplasia) of adipocyte precursors [21]. The expansion of the adipose organ increases its endocrine and secretory functions, leading to the activation of multiple pathways that may promote carcinogenesis. On the contrary, regular exercise can modify the morphology and biochemical properties of the white adipose tissue such as an increase in mitochondrial expression (browning/beiging), a reduction in the size of the adipocyte, decreased lipid content, with resultant decrease in adiposity [22,23]. A meta-analysis showed that intentional weight loss through bariatric surgery may reduce the risk of cancer [24].

## 3. Obesity, Chronic Inflammation, and Prostate Cancer

**Figure 2:**
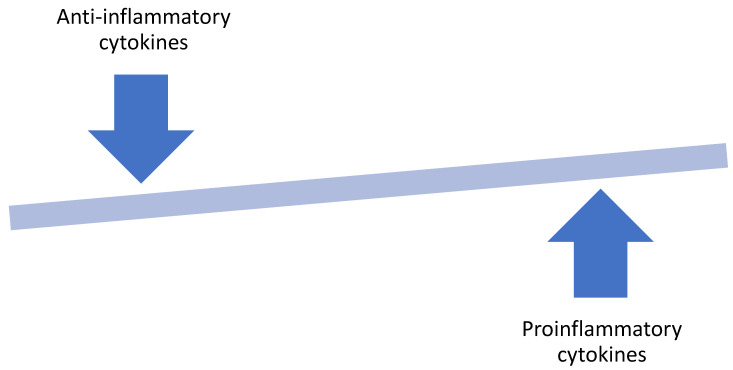
The hallmark of obesity is a shift in the adipose tissue homeostasis that favors the production of pro-inflammatory cytokines.

Inflammation of the adipose tissue caused by excess nutrient and adipose injury may be one of the crucial mechanisms of obesity-associated oncogenesis. Excess nutrients in obesity activates metabolic signaling pathways, including c-Jun N-terminal kinase (JNK), nuclear factor ĸ B (NFĸB), and protein kinase R, which leads to the activation of inflammatory cytokines, resulting in a low-grade inflammatory response [25]. Adipocyte hyperplasia and hypertrophy in obesity cause remodeling of the extracellular matrix with fibrosis in the adipose tissue microenvironment. An expanding adipose tissue outgrows its blood supply leading to hypoxia and adipocyte stress. These changes lead to an increase in free fatty acids, resulting in changes in adipokines production. The adipocytes and the existing adipose tissue macrophages secrete chemokines such as CCL2, CCL3, and RANTES/CCL5 that attracts more macrophages into the white adipose tissue [25]. In addition to the expansion of the macrophage population in the white adipose tissue, there is also a shift from an anti-inflammatory M2-like phenotype of the macrophages seen in lean white adipose tissue to the pro-inflammatory M1-like phenotype seen in obese white adipose tissue (Figure 2) [25]. This cascade of events triggers the activation of adipocyte-derived hypoxia-inducible factor (HIF-1α), and inflammatory cytokines such as tumor necrosis factor alpha (TNF-α), interleukin 6, (IL-6), interleukin 8 (IL-8) and monocyte chemotactic protein 1 (MCP-1) [26,27,28].

Although the role of chronic low-grade systemic inflammation (endocrine signaling) in carcinogenesis has been well documented, the knowledge about paracrine signaling is evolving. The prostate gland is surrounded by a periprostatic adipose depot. It is becoming more apparent that this adipose depot plays an important paracrine role in prostate cancer progression and aggressiveness. van Roermund et al. found a significant association between periprostatic fat density measured on computed tomography (CT) and having a high-risk of prostate cancer [29]. This finding has been corroborated by multiple other studies that used magnetic resonance imaging to measure the periprostatic adipose tissue thickness [30,31,32]. There is evidence of higher level of inflammatory cytokines within the periprostatic adipose tissue. There is also evidence of crosstalk between the periprostatic adipocytes and prostate cancer cells. Finley et al. found a higher level of IL-6 in periprostatic adipose tissue conditioned medium [33]. The study also showed that periprostatic adipose tissue derived IL-6 correlated with pathological grade of prostate cancer and there was greater phosphorylation on STAT3 with high grade tumors. A study by Laurent et al. showed that an adipocyte-derived chemokine, CCL7, diffuses from the periprostatic adipose tissue depot into the peripheral zone of the prostate gland to promote extraprostatic migration of CCR3-expressing prostate cancer cells [34]. Inhibition of the CCR3/CCL7 axis totally abrogated the migration, thus underscoring the therapeutic potential of this axis.

### 3.1. HIF-1α

Biological functions such as angiogenesis, cell proliferation, apoptosis, inflammation, and insulin resistance are modulated by hypoxia through the HIF‑1 complex [35]. The HIF‑1 complex consists of a heterodimer pair of HIF-1α and HIF-1β. The HIF-1 complex binds to promoter of hypoxia-responsive genes while interacting with other transcription factors such as p300 and redox effector factor 1. The transcriptional activity of HIF-1 complex is determined by the expression of the HIF-1α subunit, which is over-expressed in preneoplastic prostate lesions and has emerged as an important transcription factor in prostate carcinogenesis [36]. Zhong et al. found that HIF-1α was over-expressed in several tumor types including colon, breast, gastric, lung, skin, ovarian, pancreatic, renal, and prostate cancers compared to their respective normal tissues. HIF-1α expression correlated with aberrant p53 accumulation and cell proliferation [37].

Contrary to its effect in tumor tissue, HIF-1α is unable to induce an effective angiogenic response in adipose tissue hypoxia. Instead, it activates alternative transcriptional pathways that culminate in extracellular matrix fibrosis. There is evidence to suggest that some of these pathways include restriction of beta-oxidation of fatty acids through transcriptional suppression of SIRT2 NAD+ dependent deacetylase [38]. It also causes malfunctioning of enzymes such as lysyl oxidase and prolyl-4-hydroxylase that process proteins needed for extracellular matrix collagen synthesis [39]. These processes cause altered composition of the extracellular matrix with excessive deposition of fibrillar elements like collagens I, II, and IV, ultimately leading to metabolic and structural dysregulation and fibrosis. The paradoxical effect of HIF-1α in adipose tissue was further confirmed in a mouse model by Sun et al. [40]. Selective inhibition of HIF-1α with PX-478 led to a reduction in fibrosis and inflammatory infiltrate in the adipose tissues of mice fed with a high fat diet. It also increased their energy expenditure and reduced their weight gain.

### 3.2. TNF-α

TNF-α is an inflammatory cytokine that is involved in cell signaling and produced mainly by macrophages but can also be produced by other types of cells. It is a type II transmembrane protein with signaling potential as a membrane-integrated protein or a soluble cytokine released after proteolytic cleavage [41]. It has a wide range of activities in inflammatory and immune responses. TNF-α exerts its effect by binding to either cell membrane receptor TNFR-1 or TNFR-2, which are also known as p55 and p75, respectively. These receptors belong to the TNF receptor superfamily, which include FAS, CD40, CD27, and RANK [42]. TNFR-1 is expressed in most cells, while TNFR-2 is mainly found in hematopoietic cells. Activation of TNFR-1 and TNFR-2 leads to a cascade of cell signaling events that include the release of other inflammatory cytokines (IL-6, IL-8, and GM-CSF), expression of endothelial adhesion molecules and chemokines, and recruitment of leukocytes to target sites [43]. These events are needed for the maintenance of the immune system, inflammation, and host defense. In fact, TNF-α was named based on its ability to induce necrosis of transplanted methylcholanthrene-induced sarcoma in mice by triggering apoptosis of tumor endothelial cells [44,45]. Contrary to the anti-tumor effects of TNF, it is now clear that TNF-α is also involved in pathologic processes such as chronic inflammation, autoimmunity, and malignancy [46,47]. In a mouse melanoma model, TNF-α production induced TNFR-1 dependent death of CD8+ T-cells, thus limiting the accumulation of these tumor infiltrating lymphocytes in the tumor microenvironment. Blockade of the TNF-α/TNFR-1 signaling axis led to an increase in the proportion of melanoma-specific CD8+ T-cells in the tumor and delayed tumor growth [48]. Epithelial to mesenchymal transformation promotes stemness, invasiveness, and metastatic potential in oncogenesis. TNF-α was shown to induce epithelial to mesenchymal transformation and promote tumorigenesis in a mouse model using human renal cell carcinoma cell lines [49]. TNF-α suppressed E-cadherin, upregulated vimentin, and increased the expression of MMP9, which promotes cellular migration and invasiveness. TNF-α inhibited GSK-3β activity through the PI3K/Akt signaling pathway. Targeted inhibition of PI3K/Akt reactivated GSK-3β and suppressed TNF-α-associated epithelial to mesenchymal transformation in renal cell carcinoma cells. In contrast, activation of GSK-3β suppressed TNF-α-mediated epithelial to mesenchymal transformation, anchorage-independent growth on soft agar, and enhanced tumorigenesis in vivo [49]. TNF-α involvement in epithelial to mesenchymal transformation has been shown in other types of cancer [50,51].

### 3.3. IL-6

IL-6 is a cytokine that was originally identified as a T-cell lymphokine involved in the final differentiation of B-cells into antibody producing cells. It has both pro-inflammatory and anti-inflammatory properties. It is produced by multiple types of cells and regulates immunologic response, hematopoiesis, inflammation, and oncogenesis. The biologic effects of IL-6 are mediated through the IL-6 receptor system that comprises IL-6R and gp130 signal transducer [52]. The receptor system exists as a membrane-bound IL-6R/gp130, membrane-bound gp130, and soluble IL-6R. The classic cis-signaling occurs when IL-6 binds to the membrane-bound IL-6R/gp130 on target cells. Trans signaling happens when IL-6 binds to soluble IL-6R and the resulting IL-6/IL-6R complex binds to gp130 on target cells. The ligand/receptor complex in cis- or trans- signaling activates the intracellular JAK/STAT signaling pathway that modulates the immune system, cell division and death, and tumorigenesis. There is a plethora of evidence that IL-6 may be involved in prostate cancer development and progression. IL-6 can promote the proliferation of prostate cancer and inhibit apoptosis via the JAK/STAT pathway through the extracellular signal-regulated kinase 1 and 2 (ERK1/2)-mitogen activated protein kinase (MAPK) pathway, and the PI3K pathway [53]. The expression of IL-6 and its receptor were investigated in radical prostatectomy specimens from prostate cancer patients. IL-6 and IL-6R were found in benign prostatic epithelium, but there was over-expression of both in adjacent pre-malignant high-grade prostatic intraepithelial neoplasm (HGPIN) lesions as well as in the malignant prostate tumor tissues [54]. Serum levels of IL-6 were elevated in treatment-naïve patients with castration-resistant and metastatic prostate cancer. The levels also correlate with prostate cancer morbidity, mortality, and response to therapy [53,55]. IL-6 promoted the growth of androgen-sensitive prostate cancer in vivo and in vitro through the activation of the androgen receptor [56]. The effect was reversed by the administration of either the non-steroidal anti-androgen, bicalutamide, or the inhibitor of the MAPK pathway, PD98059.

### 3.4. IL-8

IL-8 (CXCL8), a member of the CXC family of chemokines, is a cytokine produced by various cells including macrophages in response to inflammation. It is a chemotactic factor that attracts specifically neutrophils to sites of infection or inflammation where it induces degranulation and phagocytosis. IL-8 is encoded by the CXCL8 gene and it is produced as a precursor of 99 amino acids [57,58]. The precursor then undergoes cleavage into its various isoforms, of which the 72 amino acid peptide is the most predominant. The biologic effects of IL-8 are mediated through two cell-surface, G protein-coupled receptors (CXCR1 and CXCR2). IL-8 signaling activates multiple signaling pathways with diverse effect on downstream targets. Given the wide expression of IL-8 and its receptors on cancer cells, endothelial cells, and tumor associated macrophages, IL-8 signaling promotes angiogenesis, and increases the proliferation, migration, and survival of cancer cells [59,60]. Using RNA in situ hybridization technique to localize interleukins (IL1β, IL-6, IL-8, and IL-10 mRNA) in low-grade and high-grade prostate cancer in African American men and European American men, Maynard et al. found that IL-8 was the most abundantly expressed and there was no difference between races [61]. There was a greater expression of IL-8 in higher Gleason grade (grades 4 and 5) prostate cancer compared to lower Gleason grade (grades 1–3) prostate cancer. Interestingly, the study also found a higher expression of IL-8 in inflamed benign regions of the prostate tissue, a region that may be more susceptible to tumorigenesis. Finally, IL-8 expression in LNCaP cell lines was associated with the loss of androgen receptor but not with castration resistance. The findings of this study suggest that IL-8 may be involved in the initiation and aggressiveness of prostate cancer.

### 3.5. MCP-1

MCP-1 (CCL2) is a member of the CC-motif chemokine family, and it is produced by a variety of cells constitutively or after induction by oxidative stress, cytokines, or growth factors [62]. Some of these cells include fibroblasts, endothelial, epithelial, smooth muscle, mesangial, astrocytic, monocytic, and microglial cells. MCP-1 is a chemotactic factor for monocytes and other immune cells such as natural killer cells and memory T lymphocytes. Its biological effects are mediated through its receptor, CCR2. Unlike MCP-1, which is universally expressed, the expression of CCR2 is limited to certain types of cells. The two alternatively spliced forms of CCR2 are CCR2A (the major isoform), which is expressed by mononuclear cells and vascular smooth muscle, and CCR2B, which is expressed by monocytes and activated natural killer cells [63]. CCR2 has both pro-inflammatory and anti-inflammatory effects. The pro-inflammatory effects are dependent on antigen presenting cells and T cells, while the anti-inflammatory effects are dependent on regulatory T cells. The ability of MCP-1 to induce angiogenesis is based on its chemoattractant effect on monocytes and its induction of VEGF-A gene expression [64]. MCP-1 and VEGF have been shown to be highly expressed in multiple types of cancers, and their expression has been shown to correlate with infiltration by tumor-associated macrophages, angiogenesis, and poor survival [65]. Over-expression of MCP-1 and CCR2 has been observed in both primary and metastatic prostate cancer cells [66]. Aggressive cancer cells express higher levels of CCR2 in comparison to less aggressive or benign prostatic cells. Recombinant human MCP-1 induced dose-dependent prostate cancer cell proliferation by activating the PI3K/Akt pathway. Activation of the Akt pathway provides cancer cells with a survival advantage through the upregulation of survivin [66]. MCP-1 may also play a role in bone metastasis in prostate cancer through its involvement in the differentiation and maturation of osteoclasts, which require the presence of M-CSF and RANKL. In an in vitro study, the suppression of human osteoclast differentiation by GM-CSF even in the presence of M-CSF and RANKL was reversed by the addition of MCP-1 [67]. PTHrP secreted by cancer cells acts like PTH by up-regulating RANKL and MCP-1 thereby promoting osteoclastogenesis [68].

## 4. Obesity, Adipokines, and Prostate Cancer

Adipokines are bioactive molecules with paracrine and endocrine effects that are produced by the adipose tissue. They have an array of effects in different organs and the pattern of adipokine production can reflect the adipose tissue function and status [69]. A number of these molecules have been described in the medical literature. A summary of the potential role of some of them in prostate carcinogenesis is provided below.

### 4.1. Leptin

Leptin is a polypeptide adipokine that is mainly secreted by white adipose tissue and it is involved in satiety, energy expenditure, and body weight [70]. Obesity is associated with higher circulating leptin levels. The biologic effects of leptin are mediated through leptin receptors (ObR) that are expressed in the brain and peripheral tissues in several isoforms. ObRa isoform plays an important role in transporting leptin across the blood–brain barrier, while the ObRb isoform, which is expressed in the hypothalamus, mediates signal transduction [71]. Leptin serum concentration positively correlates with body fat [72]. Leptin regulates adiposity by exerting a negative feedback effect on energy intake. However, most persons with diet-induced obesity develop leptin resistance. Hyperleptinemia promotes chronic low-grade inflammation by enhancing T-cell and macrophage activation and stimulating the production of inflammatory cytokines. In DU145 and PC-3 cell lines, leptin was shown to induce proliferation and migration of the prostate cancer cells through the PI3K and MAPK pathways [73,74]. Using LNCaP, DU145, and PC-3 cell lines, Noda et al. showed that long-term exposure to leptin increases proliferation, migration, and invasion of prostate cancer cells through the inactivation of FOXO1. The increased phosphorylation of FOXO1 was achieved through PI3K signaling. Leptin also increases ObR expression and enhanced Akt phosphorylation constitutively [75]. Although several in vitro studies have suggested an association between leptin and prostate cancer, the findings of epidemiological studies have been inconsistent.

### 4.2. Adiponectin

Adiponectin is an anti-inflammatory hormone that also regulates glucose and lipid metabolism through its insulin-sensitizing activities. In the circulation, adiponectin exists in different isoforms, which include trimer, hexamer, and high molecular weight multimers [76]. The different isoforms induce different biologic activities that are mediated through any of the three adiponectin receptors, which are adiponectin receptor subtype 1 (AdipoR1), adiponectin receptor subtype 2 (AdipoR2), and T-cadherin [76]. Unlike leptin, the plasma level of adiponectin is inversely related to body fat, being lower in the obese compared to the non-obese state.

Adiponectin is a well-recognized anti-inflammatory adipokine that has been shown to inhibit B cell differentiation in vitro and promote the expression of anti-inflammatory cytokines such as IL-10 and IL-1RA but suppresses the production of pro-inflammatory cytokine, IFN-γ [77]. In contrast, pro-inflammatory cytokines, which are highly produced in obesity, directly inhibit adiponectin transcription. The increase in visceral adipose tissue in obesity tilts this balance in favor of the pro-inflammatory cytokines such as IL-6, IL-8, TNFα, and leptin (Figure 2). These cytokines may facilitate crosstalk between the pre-tumoral epithelial cells and the adipocyte within the visceral adipose tissue microenvironment leading to tumorigenesis. Substantial evidence is in support of this in breast cancer pathogenesis. This can be explained by the fact that the breast is about 80% composed of adipose tissue, and thus the mammary epithelial cells are in close proximity to adipocytes and other inflammatory cells. This cellular juxtaposition exposes the mammary epithelial cells to changes in cytokine levels within the adipose tissue microenvironment. Similar structural arrangement is probably applicable in prostate cancer due to the location of the prostate within the periprostatic fat. Several studies have shown that the level of circulating adiponectin is lower in patients with different types of malignancies. Wei et al. performed a meta-analysis of 107 studies (including 13 prostate cancer studies) to determine the association between circulating adiponectin levels and cancer. The pooled analysis revealed that circulating levels of adiponectin and high molecular weight adiponectin were lower in cancer patients compared to their controls [78]. Goktas et al. also found that the plasma levels of adiponectin were significantly lower in patients with prostate cancer compared to patients with benign prostatic hypertrophy as well as controls. Additionally, plasma adiponectin levels had an inverse association with the prostate specific antigen levels and the Gleason scores [79]. This was further proven in a prospective analysis of the Physicians’ Health Study that found no association between adiponectin concentration and overall risk of prostate cancer, but men with higher adiponectin concentrations had a lower risk of developing high grade prostate cancer and lower risk of dying from the disease [80].

### 4.3. Visfatin

Visfatin is also known as pre-B-cell colony-enhancing factor (PBEF) and nicotinamide phosphoribosyltransferase (NAMPT). It is a rate-limiting enzyme involved in the regeneration of nicotinamide adenine dinucleotide (NAD^+^) from nicotinamide. Hence, it has an important role in many cellular functions by regulating NAD^+^-dependent SIRT1 deacetylase [81]. There are conflicting reports in the medical literature on the association between visfatin and obesity. While some studies have reported increased plasma visfatin levels in various cohorts of obese patients, others have reported decreased or no change in visfatin levels. A meta-analysis that was designed to address this controversy showed that plasma concentrations of visfatin were elevated in patients with obesity [82]. Visfatin has proinflammatory properties and has been shown to stimulate endothelial proliferation and capillary tube formation via the upregulation of VEGF and matrix metalloproteinases (MMP-2 and MMP-9) mediated by MAPK/PI3K-Akt/VEGF signaling pathways [83]. Visfatin was also shown to reduce apoptosis in human umbilical vein endothelial cells (HUVECs) [83]. Visfatin, along with SIRT1, is over-expressed in human prostate cancer and over-expression of visfatin increases prostate cancer cell resistance to oxidative stress [81]. Conversely, inhibition of visfatin was shown to significantly suppress PC3 and LNCaP prostate cancer cell growth, colony formation, and invasion, leading to dramatic apoptosis.

### 4.4. FGF21

Fibroblast growth factor 21 (FGF21) is a polypeptide with 210 amino acid residues. It is one of the three members (FGF19/15, FGF21, and FGF23) of the FGF family that have emerged as endocrine factors involved in metabolic regulation [84]. The serum levels of FGF21 are significantly higher in obesity than in the lean state. The levels also correlate positively with adiposity, fasting insulin level, and triglyceride [84]. FGF21 stimulates insulin-independent glucose uptake in adipocytes through the enhancement of GLUT1 expression, and it is also involved in thermogenesis by enhancing the expression of UCP1 and other thermogenic genes in adipose tissue [84]. The expression of FGF12 is induced by cold exposure and beta-adrenergic stimulation in brown adipose tissue and thermogenic competent white adipose tissues [85]. The role of FGF21 in prostate carcinogenesis is still evolving. A study by Dai et al. showed lower expression of FGF21mRNA and protein in prostate cancer cell lines (LNCaP, PC3, DU145, 22Rv1) than prostate epithelial cells (RWE-1) using RT-PCR and Western blot [86]. FGF21 expression by immunohistochemistry was also lower in clinical prostate cancer tissues than benign prostatic hypertrophy. Increased expression of FGF21 in p-FGF21 transfected LNCaP cells inhibited LNCaP cell proliferation, clone formation, migration, and invasiveness. FGF21 overexpression attenuated high glucose-induced LNCaP cell proliferation and promoted apoptosis. FGF21 promoted autophagy through the inhibition of the PI3K/Akt/mTOR signaling pathway [86].

### 4.5. BMP

Bone morphogenetic proteins (BMPs) are a group of polypeptide growth factors that were so named because of their ability to induce ectopic bone formation. Newer information shows that BMPs have other biologic functions during embryonic development and in postnatal homeostasis of various organs and tissues, by modulating cell lineage commitment, morphogenesis, differentiation, proliferation, and apoptosis [87]. BMPs are produced by several cells in the body, including the adipocyte. BMP2 and BMP4 have structural similarities and are pro-adipogenic. While more than a dozen BMPs that have been discovered structurally belong to the transforming growth factor β T(GFβ) superfamily, BMP1 is a metalloprotease. The actions of BMP are mediated through two types of transmembrane serine/threonine kinase receptor (type I and type II), and intracellular downstream signaling is through BMP-specific SMAD proteins [87]. The preponderance of osseous metastases in prostate cancer has generated interest in the potential role of BMPs in the interactions between prostatic tumor cells and bone. However, the findings have been conflicting. BMP2, BMP4, and BMP7 are predominantly expressed in normal prostatic tissue and their expressions tend to decline with disease progression. In contrast, BMP6 is more likely to be expressed in metastatic prostate cancer but not in non-metastatic or benign prostate tissue. The expression tends to be higher in high-grade primary tumors. The expression of BMP7 has been shown to be androgen-dependent in both mouse and human prostates [88]. In summary, the differential expression of each BMP may be due to changes in the prostate tumor cell phenotype and each may play different roles at different stages of the disease.

## 5. Obesity, Insulin-like Growth Factor, and Prostate Cancer

Excessive body fat with its attendant metabolic syndrome is characterized by insulin resistance, which necessitates increased production of insulin from pancreatic beta cells to maintain glucose homeostasis. The resultant hyperinsulinemia increases hepatic synthesis of insulin-like growth factor (IGF) and changes in the concentrations of IGF-binding proteins.

### IGF

The IGF axis is a complex system of cell surface receptors (IGF1R, IGF2R, and insulin receptor, IR), ligands (IGF-1, IGF-2), several high-affinity IGF-binding proteins (IGFBPs), several low-affinity IGFBP-related proteins (IGFBP-rPs), and IGFBP proteases [89,90]. The IGF axis controls cellular metabolism, tissue homeostasis, and cell survival through the activation of the MAPK and the PI3K-Akt signaling pathways. It is involved in the epithelial-to-mesenchymal transformation and the attainment of a malignant phenotype. The IGFBPs and IGFBP-rPs transport IGFs, modulate their half-life, and regulate their access to their receptors. Therefore, the biologic activities of IGFs are dependent on both their interaction with their receptors and the influence of the binding proteins. On the other hand, the activities of the binding proteins are regulated by the IGFBP proteases [90]. IGFBP3 is the most abundant circulating IGFBP and it competitively binds to IGF1 against the IGF1 receptor. Consequently, IGFBP3 may inhibit cell proliferation and survival, and the loss of IGFBP3 expression may contribute to drug resistance [72]. Some antiproliferative and proapoptotic actions of IGFBP3 are independent of IGF but are mediated through the interaction of IGFBP3 with retinoid X receptor, vitamin D receptor, and TGFβ/SMAD signaling pathways [90]. IGFBP-rP1 is a low-affinity IGF-binding protein that was named IGFBP7 initially. Unlike the other IGFBP, its affinity for IGF1 is 100-fold lower but it binds strongly to insulin and inhibits the phosphorylation of the insulin receptor. The expression of IGFBP-rP1 is upregulated in some cancers while it is downregulated in some others, suggesting that IGFBP-rP1 may play a dual role as promoter or suppressor of malignancy. There are conflicting reports on the expression of IGFBP-rP1 in the normal epithelium as compared to malignant prostatic tissue. While some studies have shown a gradual decrease in the expression of IGFBP-rP1 from normal to malignant prostatic epithelium, other have disproved this differential expression [91,92]. Conflicting reports also abound on the association between IGF and prostate cancer in epidemiologic studies [89,93]. In the European Prospective Investigation into Cancer and Nutrition (EPIC) study, circulating IGF1 concentration was positively associated with a significant increase in the risk of prostate cancer [93]. A meta-analysis of prospective and retrospective studies showed a positive association between IGF1 and the risk of prostate cancer but no significant association between IGF2, IGFBP1, IGFBP2, or IGFBP3 and the risk of prostate cancer [94].

## 6. Conclusions

Obesity is a pandemic of increasing proportion and an established risk factor for multiple types of malignancies, including prostate cancer. Although the underlying mechanisms are not completely understood, the imbalance between the pro-inflammatory and anti-inflammatory cytokines in the adipose tissue microenvironment, and the differential expression of certain genes may play critical roles in prostate carcinogenesis and disease proliferation. While several pre-clinical studies have provided an insight into the association between obesity and prostate cancer, the findings from epidemiological studies have been inconsistent. Therefore, further studies are needed to validate the findings from pre-clinical studies as actionable therapeutic targets if effective preventive measures and treatment agents are going to be developed.
